# Probiotics and Prebiotics: Present Status and Future Perspectives on Metabolic Disorders

**DOI:** 10.3390/nu8030173

**Published:** 2016-03-18

**Authors:** Ji Youn Yoo, Sung Soo Kim

**Affiliations:** 1 Department of Biomedical Science, Graduate School, Kyung Hee University, Seoul 02447, Korea; yoojiyoun@gmail.com; 2 Department of Biochemistry and Molecular Biology, Medical Research Center for Bioreaction to Reactive Oxygen Species and Biomedical Science Institute, School of Medicine, Kyung Hee University, Seoul 02447, Korea

**Keywords:** metabolic disorders, type 2 diabetes (T2DM), cardiovascular diseases (CVD), gut microbiota, probiotics, prebiotics

## Abstract

Metabolic disorders, including type 2 diabetes (T2DM) and cardiovascular disease (CVD), present an increasing public health concern and can significantly undermine an individual’s quality of life. The relative risk of CVD, the primary cause of death in T2DM patients, is two to four times higher in people with T2DM compared with those who are non-diabetic. The prevalence of metabolic disorders has been associated with dynamic changes in dietary macronutrient intake and lifestyle changes over recent decades. Recently, the scientific community has considered alteration in gut microbiota composition to constitute one of the most probable factors in the development of metabolic disorders. The altered gut microbiota composition is strongly conducive to increased adiposity, β-cell dysfunction, metabolic endotoxemia, systemic inflammation, and oxidative stress. Probiotics and prebiotics can ameliorate T2DM and CVD through improvement of gut microbiota, which in turn leads to insulin-signaling stimulation and cholesterol-lowering effects. We analyze the currently available data to ascertain further potential benefits and limitations of probiotics and prebiotics in the treatment of metabolic disorders, including T2DM, CVD, and other disease (obesity). The current paper explores the relevant contemporary scientific literature to assist in the derivation of a general perspective of this broad area.

## 1. Introduction

Metabolic diseases, such as type 2 diabetes (T2DM) and cardiovascular diseases (CVD), present an important social problem, considering the increasing morbidity rate in both developing and developed countries. Over the last decade, dynamic changes in dietary macronutrient ingestion and lifestyle have rapidly increased the prevalence of metabolic disorders. T2DM patients have a higher risk of CVD, the primary cause of death. Recently, scientists and nutritionists have proposed that metabolic disorders might result from an alteration in gut microbiota composition [1,2]. *Bacteroidetes* and *Firmicutes* are dominant (>90% of the total microbial population) in human intestine and play a significant role in nutrient absorption, mucosal barrier fortification, xenobiotic metabolism, angiogenesis, and postnatal intestinal maturation. Diet controls the composition of these bacteria, which are crucial in the development of metabolic disorders [3,4,5,6,7].

The term “probiotic” originates from the Greek word meaning “for life” [8]. In 1989, Fuller defined the term probiotic as “a live microbial feed supplement which beneficially affects the host animal by improving its intestinal balance” [8]. In 1995, Gibson *et al.* defined prebiotics, on the other hand, as “a non-digestible food ingredient that beneficially affects the host by selectively stimulating the growth and/or activity of one or a limited number of bacteria in the colon” [9]. A long history of human consumption of probiotics (particularly *lactic acid bacteria* and *bifidobacteria*) and prebiotics exists, either as natural components of food or as fermented foods. In 76 B.C., the Roman historian Plinius recommended the ingestion of fermented milk products to a patient who had gastroenteritis [10]. Probiotics and prebiotics began to blossom in the late 1800s and early 1900s. Subsequently, Metchnikoff noticed health effects stemming from the alteration of the intestinal microbial balance, and he proposed that the consumption of yogurt containing *Lactobacillus* would result in a decrease in toxin-producing bacteria in the gut and an increase in the longevity of the host [11,12]. In 1900, Tissier recommended the addition of *bifidobacteria* to the diet of infants suffering from diarrhea, claiming that *bifidobacteria* superseded the putrefactive bacteria that caused the condition [13,14]. Since then, numerous scientists have noticed that bacteria in the colon produce many different types of compounds that maintain both positive and negative effects on gut physiology, as well as other systemic influences [15,16,17]. As an example, short-chain fatty acids (SCFAs) are produced by the fermentation of bacteria, when the bacteria in the colon metabolize proteins and complex carbohydrates. These SCFAs may decrease the risk of developing metabolic disorders due to the increasing demand of cholesterol for *de novo* synthesis of bile acids [18]. Probiotics and prebiotics are considered to be alternative supplements against metabolic disorders, as the manner of their action is thought to be based largely on a modulation of the composition and function of the intestinal microbiota. Several studies have shown that probiotics and prebiotics play an important role in the amelioration of T2DM and CVD [19,20,21]. A number of researchers studied the potential of food-grade bacteria for treating or preventing diabetes. The studies indicated that certain probiotics (*L. lactis, bifidobacteria*) secrete an insulin analog and promote the expected biological effect on target adipocytes both in human and in animal subjects [22,23]. Accumulating evidence suggests that supplementation of probiotics and prebiotics could have preventative and therapeutic effects on CVD due to a reduction in total serum cholesterol, low-density lipoprotein (LDL-cholesterol), and inflammation [20,24]. This highlights a growing recognition of the role of probiotics and prebiotics in modulating the metabolic activities of the human gut microbiota and regulating the immune system, in turn improving the host’s health.

We analyze the current knowledge of the molecular mechanisms by which probiotics and prebiotics participate in host functions that affect the prevention and treatment of metabolic disorders, including T2DM, CVD, and obesity. The current review focuses on the important functions of probiotics and prebiotics through relevant contemporary studies to assist in the derivation of a general perspective of this broad area.

## 2. Gut Microbiota Compositions and Metabolic Disorders

Interactions between the gut microbiota and the host’s overall health begin at birth, and the nature of microbial diversity changes throughout the host’s life. The interaction of gut epithelial cells with microbes and their metabolites is a key mediator of the cross-talk between the gut epithelium and other cell types [25]. Additionally, this interaction assists in maturation of the intestinal epithelial layer, the enteric nervous system, the intestinal vascular system, and the mucosal innate immune system. Human gut microbiota are strongly involved in diverse metabolic, nutritional, physiological, and immunological processes, and changes in the composition of the gut microbiota directly influence the host’s health [1,26]. Although early intestinal microbiota studies focused on only a minority of bacteria species and their functions, recent researchers have discovered more than 1100 bacteria species and were able to analyse their functional properties as related to certain disease states, such as T2DM, CVD, obesity and cancer, because of the development of advanced techniques, such as DNA-based analyses [27]. In particular, changes of gut microbiota composition are strongly associated with increased adiposity, β-cell dysfunction, metabolic endotoxemia, systemic inflammation, and oxidative stress associated with T2DM [28].

Intestinal microbiota can affect host adiposity and regulate fat storage which, in some cases, can contribute to obesity [3,29]. The change in intestinal microbiota and the reduced bacterial diversity were also observed in obese conditions. For example, Ley *et al.* demonstrated a significant relationship between gut microbiota composition and obesity. This study showed that the number of *Firmicutes* increased while the number of *Bacteroidetes* decreased in obese mice compared to lean mice [30]. Furthermore, other studies revealed that transplantation of microbiota from obese mice into germ-free mice, despite reduced food intake, significantly increased adipose tissues compared to transplantation of microbiota from lean mice [31]. Larsen *et al.* also demonstrated that the proportions of *Bacteroidetes* to *Firmicutes*were significantly and positively associated with reduction of glucose tolerance. They showed that microbiome diversity was not different between T2DM and non-DM patients, but the composition and function were different, including butyrate-producing bacteria and opportunistic pathogens [32]. The change of these bacteria compositions increases susceptibility to infections, immune disorders, inflammation, oxidative stress and insulin resistance, events that are mediated by metabolic endotoxemia, which involves exposure to noxious intestinal products, particularly lipopolysaccharides (LPS) [33]. LPS is a component of the gram-negative bacteria’s cell wall. LPS binds to toll-like receptor-4 (TLR4) on endothelial cells, monocytes,and macrophages. The reaction initiates an inflammatory response and oxidative stress, leading to the activation of NF-κB and AP-1. These activations produce pro- inflammatory cytokines, chemokines, adhesion molecules and reactive oxygen species (ROS), which can cause endothelial damage and dysfunction. For example, trimethylamine N-oxide (TMAO) contributesto the development and progression of cardiovascular disease and the early detection of myocardial injury [34]. TMAO, an oxidation product of trimethylamine (TMA), is a relatively common metabolite of choline in animals [35]. Tang *et al.* validated that increased TMAO levels are associated with increased risk of incidence of major adverse cardiovascular events in a large independent clinical cohort (*n* = 4007). According to the study, people in the highest quartile of circulating TMAO levels had a 2.5-fold increased risk of having a major adverse cardiac event, when compared to those in the lowest quartile [36]. Furthermore, TMAO levels were dose-dependently related to obesity and insulin resistance in animal studies [37]. Although the mechanisms by which circulating TMAO promotes CVD are currently unclear, there is a possible hypothesis of cardiovascular physiology. Expression of scavenger receptors (CD36 and SR-A1) on macrophages and foam cell formation were increased by supplementation of TMAO in normal chow diet mice [38]. Furthermore, supplementation of TMAO reduces reverse cholesterol transport in macrophage, which would be predicted to advance atherosclerosis [39]. Although supplementation of TMAO clearly influences multiple steps of both forward and reverse cholesterol transport, the underlying molecular mechanisms behind these observations remain unclear. Therefore, further study should be performed to elucidate how circulating TMAO levels are sensed to elicit pathological responses and to explain mechanisms by which TMAO promotes CVD.

Numerous studies also support the theory that gut microbiota can influence host immune functions. Gut microbiota cooperate with the host immune system through an extensive array of signalling pathways, which involve many different classes of molecules and extend beyond the immune system. These immune-mediated signalling processes are directly associated with chemical interactions between the microbe and the host.

## 3. Probiotics

The definition of a probiotic is “a live microbial feed supplement which beneficially affects the host animal by improving its intestinal balance” [40]. The initial concept of probiotics originated from the work of Metchnikoff at the beginning of the 20th century. Subsequently, Shaper *et al.* (1963) and later Mann (1974) observed a reduction in serum cholesterol after consumption of copious amounts of milk fermented with wild *Lactobacillus* and/or *Bifidobacterium* [41,42]. Probiotics have been investigated as a potential dietary supplement that can positively contribute to an individual’s health [43]. These health benefits are not limited to the intestinal tract, but also include amelioration of systemic metabolic disorders, such as T2DM and CVD.

Since probiotics have been recognized as a key health promoter thought to stem from the modulation of host immune responses [44], earlier studies have mainly focused on the relationship between probiotics and immune diseases, such as atopic dermatitis and inflammatory bowel disease. Intestinal bacteria, including *Lactobacilli* and *Bifidobacterium*, can cross the intestinal mucous layer and stimulate phagocytic activities in the spleen or in other organs for many days [45]. Proliferative responses of spleen cells to concanavalin A (a T-cell mitogen) and lipopolysaccharide (a B-cell mitogen) were significantly enhanced in mice supplied with *Lactobacillus rhamnosus*, *Lactobacillus acidophilus*, or *Bifidobacterium.* Despite administration of these probiotics, the mice did not exhibit any significant increase in interleukin-4 production by spleen cells nor peripheral blood leucocytes. Instead, spleen cells from mice that consumed these probiotics produced significantly higher amounts of interferon-γin response to stimulation with concanavalin A, compared to cells from the control animals [46]. 

Several studies have demonstrated that patients with T2DM have a significantly lower number of bacteria that produce butyrate when compared to healthy people. Larsen *et al.* showed an association between T2DM and compositional changes in the intestinal microflora. In particular, they demonstrated a considerably lower proportion of phylum *Firmicutes* and *bifidobacteria* in T2DM patients than in non-diabetic individuals [32,47]. Interestingly, several studies have revealed that probiotics and prebiotics might maintain the potential to improve lipid profiles, including the reduction of LDL-cholesterol, serum/plasma total cholesterol, and triglycerides or increment of high-density lipoprotein (HDL-cholesterol) in the context of treating CVD [22,44,48,49,50,51,52]. Previous studies have proven that the administration of certain probiotics can promote short-chain fatty acids (SCFAs) that alter secretion of incretin hormones and attenuate cholesterol synthesis [53].

## 4. Prebiotics

A prebiotic was first defined as “a non-digestible food ingredient that beneficially affects the host by selectively stimulating the growth and/or activity of one or a limited number of bacteria in the colon, and thus improves host health” [9]. Subsequently, Roberfroid stated that “A prebiotic is a selectively fermented ingredient that allows specific changes, both in the composition and/or activity in the gastrointestinal microflora that confers benefits upon host well-being and health.” [9,54]. Gibson *et al.* examined three criteria, namely: (a) resistance to gastric acidity, hydrolysis by mammalian enzymes, and gastrointestinal absorption; (b) fermentation by intestinal microflora; and (c) selective stimulation of the growth and/or activity of intestinal bacteria associated with health and well-being [55]. Currently, the prebiotics that fulfill these three criteria are fructooligosaccharides, galactooligosaccharides, lactulose, and non-digestible carbohydrates. The non-digestible carbohydrates include large polysaccharides (inulin, resistant starches, cellulose, hemicellulose, pectins, and gums), some oligosaccharides that escape digestion, and unabsorbed sugars and alcohols. Most prebiotics, including fructooligosaccharides and inulin, are digested by *bifidobacteria* and stimulate the growth of their colonies. These bacteria influence homeostasis of intestinal cells and inhibit the growth of pathogenic bacteria [56,57,58].

SCFAs, such as acetic acid, propionic acid, and butyric acid, are the essential end-products of carbohydrate metabolism. Fermentation of carbohydrates represents a major source of energy for epithelial cells in the colon [59]. SCFAs reduce the development of gastrointestinal disorders, cardiovascular diseases, and cancers by inducing apoptosis (programmed cell death) [18,60]. Furthermore, prebiotics could stimulate the immune system, produce Vitamin B, inhibit pathogen growth, and lower blood ammonia. They also appear instrumental in promoting cell differentiation, cell-cycle arrest, and apoptosis of transformed colonocytes by inhibiting the enzyme histone deacetylase and decreasing the transformation of primary to secondary bile acids [9]. Moreover, SCFAs decrease glucagon levels in a dose-dependent manner, improve glucose tolerance, and activate glucagon-like peptide1 (GLP-1), which can stimulate the elevation of insulin production and increase insulin sensitivity [61,62]. Thus, administration of prebiotics probably plays a regulatory role in modulating endogenous metabolism.

## 5. Effects of Probiotics and Prebiotics on T2DM

Over recent decades, an abundance of evidence has emerged to suggest a close link between T2DM, CVD, and inflammation. Insulin plays an important role in the regulation of glucose homoeostasis and lipid metabolism. The failure of target organs to respond to the normal action of insulin is termed *insulin resistance*, which in turn often results in compensatory hyperinsulinemia. This hyperinsulinemia leads to an array of metabolic abnormalities thought to constitute the pathophysiologic basis of metabolic syndrome which can lead to CVD and coronaryheart disease [63].

Moreover, an excess accumulation of visceral fat leads to insulin resistance. In addition, this excess causes a chronic low-grade inflammation characterized by increased macrophage infiltration and pro-inflammatory adipokine production. Pro-inflammatory adipokines obstruct the insulin-signaling pathway in peripheral tissues and promote the development of insulin resistance [63,64]. These data indicate that T2DM is associated with a state of chronic low-level inflammation that leads to the development of CVD. The molecular and cellular underpinnings of obesity-induced inflammation and the signaling pathways at the intersection of metabolism and inflammation contribute to T2DM and CVD [51,52,65].

SCFAs maintain important functions in T2DM patients. Interestingly, some studies have found that the number of SCFAs producing bacteria were significantly lower in people with T2DM. These SCFAs not only bind to G-protein coupled receptors (GPCRs), but also cause the exhibition of various biological effects. For example, SCFAs promote secretion of GLP-1, one of the major incretin hormones primarily synthesized by entero-endocrine L-cells. This hormone inhibits glucagon secretion, decreases hepatic gluconeogenesis, improves insulin sensitivity, and enhances central satiety, resulting in weight loss [66]. Furthermore, some evidence indicates that SCFAs may directly prevent low-grade inflammatory response, as bacteria actively translocate from the intestines into the mesenteric adipose tissue (MAT) and the blood. Amar *et al.* proved that certain probiotics (e.g., *Bifidobacterium animalis* subsp. *lactis* 420) could reverse the low-grade inflammatory response by reducing mucosal adherence and bacterial translocation of gram-negative bacteria from the *Enterobacteriaceae*. As a result, probiotics may attenuate adipose tissue inflammation and several features of T2DM [48]. Asemi *et al.* demonstrated the effects of oral supplements of probiotics on metabolic profiles, high sensitivity C-reactive protein (hs-CRP), and oxidative stress in T2DM. In this randomized, placebo-controlled, and parallel designed study, they utilized an oral supplement comprising seven viable and freeze-dried strains: *Lactobacillus acidophilus*, *Lactobacillus casei*, *Lactobacillus rhamnosus*, *Lactobacillus bulgaricus*, *Bifidobacterium breve*, *Bifidobacterium longum*, and *Streptococcus thermophilus.* The test subjects ingested the supplement for eight weeks. The results indicated that the consumption of multi-probiotics led to a meaningful reduction in fasting plasma glucose compared to the placebo group [67].

Additionally, probiotics could promote antioxidation in T2DM patients. Erythrocyte superoxide dismutase, glutathione peroxidase activities, and total antioxidants increased in the group supplemented with probiotic yogurt compared to the control group [68]. Administration of *Lactobacillus acidophilus* and *Lactobacillus casei* with dahi (yogurt in the Indian subcontinent) significantly suppressed streptozotocin (STZ)-induced oxidative damage in pancreatic tissues by inhibiting the lipid peroxidation and nitric-oxide formation [69]. Yadav *et al.* also demonstrated that administration of the probiotic dahi in the diet significantly delayed the onset of glucose intolerance, hyperglycemia, hyperinsulinemia, and dyslipidemia, and decreased oxidative stress in high fructose-induced diabetic rates [70]. 

In contrast, few papers demonstrated that probiotics fail to maintain significant effects on the lipid profiles of T2DM patients. One of these studies concluded that supplementation of probiotics failed to cause significant changes in total cholesterol, LDL-cholesterol, HDL-cholesterol, triglycerides (TG), TG/LDL, or LDL/HDL ratios, following eight weeks of intervention [71,72]. Additionally, Lewis *et al.* showed that *lactobacillus acidophilus* administered to 80 hypercholesteraemic volunteers for six weeks failed to produce any significant effects of probiotics on serum blood lipid [73]. Although some studies showed no benefits of probiotics on serum lipids, numerous animal or human studies have demonstrated the benefits of probiotics and prebiotics. Hence, further studies are required to improve our knowledge of, and eliminate uncertainties regarding, probioticsand prebiotics (Table 1 and Table 2).

## 6. Effect of Probiotics and Prebiotics on CVD

Cardiovascular disease (CVD) affects blood vessels and/or the heart. CVD primarily stems from hypercholesterolemia and dyslipidemia. Particularly, a high level of LDL-cholesterol is most commonly associated with CVD. CVD represents the most prevalent cause of death in T2DM patients. The relative risk of CVD is two to four times higher in T2DM patients than in non-diabetic people. The most common lipid pattern in people with CVD consists of increased triglyceride-rich lipoproteins, high levels of LDL-cholesterol, and low levels of HDL-cholesterol.

Healthy nutrition and lifestyle intervention constitute important parts of managing CVD. Hypercholesterolemia patients may avoid the use of cholesterol-lowering drugs by practicing dietary control or through administration of probiotics and/or prebiotics. Health food supplements, such as probiotics and prebiotics, can modulate gut health and regulate the immune system through gut microbiota. Persuasive studies have shown that well-established probiotics and/or prebiotics possess hypocholesterolaemic effects in humans and animals. Nguyen *et al.* demonstrated that total serum cholesterol and triglycerides were significantly reduced in hypercholesterolaemic mice that ingested *Lactobacillus plantarum* PH04 [76]. Moreover, some studies supportedthatbuffalo milk yogurt and soymilk yogurt containing *Bifidobacterium* Bb-12 or *Bifidobacterium longum* Bb-46 were highly effective in decreasing the concentration of total cholesterol by 50.3%, LDL- cholesterol by 56.3%, and triglycerides by 51.2% compared to the levels of the control group [75,79,81]. Anderson *et al.* completed a similar study, but they utilized a different probiotic called *Lactobacillus acidophilus* L1. They showed that daily consumption of 200 g of yogurt containing *Lactobacillus acidophilus* after each dinner contributed to a significant reduction in serum cholesterol concentration compared to the placebo group [78]. Another study indicated that the combination of bacteria strains more effectively reduced total cholesterol and liver cholesterol compared to individual bacteria strains. The supplied mixed-bacteria and *Lactobacillus acidophilus* groups exhibited a 23%–57% decrease of cholesterol concentrations in the liver compared to the control group. Additionally, cholesterol concentration in the supplied mixed-bacteria group was lower than in single-bacteria supplemented groups [74].

Prebiotics may lead to hypocholesterolemia via two different mechanisms. First, lower cholesterol absorption is caused by enhanced cholesterol excretion via feces. The other mechanism is the production of SCFAs upon selective fermentation by intestinal bacterial microflora [77]. Causey *et al.* concluded that a daily intake of 20 g of inulin (longer-chain prebiotics, containing 9–64 links per saccharide molecule, fermented more slowly) significantly reduced serum triglycerides compared to the control group. They also found that serum LDL-cholesterol decreased and serum HDL-cholesterol increased following the administration of inulin compared to the control group [84]. Another study showed that when normolipidemic individuals consumed 18% of inulin on a daily basis without any other dietary restrictions, total plasma cholesterol and triacylglycerols decreased by 7.9% ± 5.4% and 21.2% ± 7.8%, respectively. Glucose tolerance tests demonstrated that inulin significantly enhanced breath H2 excretion (IAUC test 280 ± 40; placebo 78 ± 26 ppm × h), as well as fecal concentration of *Lactobacillus*-*lactate* [83]. Thus, inulin may possess lipid-lowering potential in normolipidemic people, possibly mediated by mechanisms related to colonic fermentation. The addition of inulin in the diet of rats induced higher excretions of fecal lipids and cholesterol compared to that of rats in the control group. This increased level of excretion is attributed primarily to reduced cholesterol absorption [85]. Other prebiotics, such as oligodextrans, lactose, resistant starches and their derivatives, lactoferrin-derived peptides, and *N*-acetylchitooligosaccharides have also been identified as maintaining hypocholesterolaemic effects in people with T2DM who are at high risk of developing CVD [55].

Although numerous studies have documented the cholesterol-lowering effects of probiotics and/or prebiotics in both *in vitro* and *in vivo* experiments, the effects remain controversial. Hatakka *et al.* refuted the purported hypocholesterolaemic effect of probiotics, and reported that the administration of *Lactobacillus rhamnosus* LC705 failed to influence blood lipid profiles in 38 men with mean cholesterol levels of 6.2 mmol/L after a four-week treatment period [82]. Lewis *et al.* argued that the administration of *Lactobacillus acidophilus* failed to affect any serum lipid changes [73]. Furthermore, Simonsa *et al.* showed that a supplement of *Lactobacillus fermentum* failed to significantly change plasma total cholesterol, LDL-cholesterol, HDL-cholesterol, or triglycerides [80]. Although many studies suggest that probiotics can favorably alter serum lipids, some human studies examining the benefits of probiotics on serum lipids have shown conflicting results. This may bedue to the possibility that different delivery systems may affect the experiment result. The human studies, which used capsules probiotics, did not show significant changes inserum lipids compared to fermented bacteria product. A study assumed that sufficient time was not available for the freeze-dried probiotic capsule to become metabolically fully activated before being flushed into the colon. They thought that fermented dairy products can be metabolically active when ingested, whereas freeze-dried probiotic capsules cannot because the small intestinal transit is relatively short [73]. Furthermore, during the intervention, the human studies could not control for an individual’s life style, including dietary intake, whereas animal studies could, which may be one of the possible reasons for the apparent lack of effect. Therefore, further researches are required to unequivocally establish the potential role ofprobiotics in the management of metabolic disorder (Table 1 and Table 2).

## 7. Others (Obesity)

Obesity causes low-grade inflammation and an altered composition of the gut microbiota. Some studies have attempted to identify correlations between the composition of the microbiota and the occurrence of inflammation and metabolic alterations in individuals with obesity [86,87,88]. The low-grade systemic inflammation in the obese phenotype is attenuated by peptides produced in the gut. The composition of gut microbiota affects synthesis of these peptides. One such protein is the serum amyloid A3 protein (SAA3). The gut microbiota serve to regulate SAA3 expression in the adipose tissue [89,90,91]. Expression of this peptide was considerably higher in the adipose tissue and colon of mice colonized with a normal gut microbiota from a healthy wild-type mouse when compared with germ-free mice [87]. Collectively, these findings suggest that the gut microbiota modulate the biological systems that regulate the availability of nutrients, energy storage, fat mass development, and inflammation in the host, each of which is associated with the obese phenotype [92,93]. Significantly, the number of *bifidobacteria* is inversely correlated with fat mass, glucose intolerance, and LPS level [94,95]. Furthermore, inulin-type fructans affect gut ecology and stimulate immune cell activity. They also decrease weight gain and fat mass in obese individuals [96,97,98].

## 8. Molecular Mechanisms of Action

Several hypotheses have been presented to explain how the mechanistic actions of probiotics and prebiotics, including the improvement of gut microbiota, the stimulation of insulin signaling, and the lowering of cholesterol, ameliorate the T2DM and CVD condition. Among the molecular mechanisms, the current paper focuses on SCFA receptors and bile-salt hydrolase (BSH) that are associated with regulation of insulin secretion, fat accumulation, and cholesterol levels.

Recently, two orphan GPCRs, GPR41 (known as FFAR3) and GPR43 (known as FFAR2), were found to be receptors for SCFAs, including acetate, propionate, and butyrate. FFAR2 is primarily activated by acetate and propionate, whereas FFAR3 is more often activated by propionate and butyrate [99]. Both receptors are mainly expressed in L cells, which are located along the length of the intestinal epithelium and respond directly to luminal signals [100]. FFAR2 and FFAR3 stimulate the release of GLP-1 and peptide YY (PYY), which improve insulin secretion. The expression levels of GLP-1 and PYY are often reduced in individuals with T2DM. Therefore, enhancement of GLP-1 and PYY secretion from intestinal L cells could result in beneficial effects in people with T2DM. 

Several studies have shown that a deficiency of FFAR2 decreases SCFA-induced secretion of GLP-1 both *in vitro* and *in vivo*, and enhances insulin resistance. The injectable GLP-1 mimetics are associated with good blood glucose control and a decreased incidence of hypoglycemia [100,101,102]. In addition, FFAR2 regulates energy metabolism via promotion ofleptin secretion, adipogenesis, and inhibition of lipolysis in adipose tissue and adipocytes [103]. Obesity is frequently observed in FFAR2-deficient mice on a normal diet, while overexpressed FFAR2 in adipose tissue mice remain lean, even though the mice are fed a high-fat diet. Isoproterenol-induced lipolysis is inhibited by SCFSs in a dose-dependent manner in mouse 3T3-L1 derived adipocytes [104,105]. Kimura *et al.* concluded that FFAR2 activation by SCFAs suppressed adipose-specific insulin signaling in white adipose tissues, and thus led to the inhibition of fat accumulation [105]. 

Similarly, Samuel *et al.* demonstrated that germ-free mice with or without FFAR3 were colonized by specific microbes. The results showed that PYY levels were decreased in FFAR3-deficient mice, indicating that the secretion of PYY from the intestine was regulated by SCFA-induced FFAR3 [106,107]. Moreover, FFAR3 is abundantly expressed in sympathetic ganglia. Inoue *et al.* showed that SCFA-induced FFAR3 activation resulted in increased heart rate and energy expenditure through sympathetic activation. Notably, the effects were not observed in FFAR3-deficient mice. FFAR3 also directly promotes noradrenalin release from sympathetic neurons [108,109]. In contrast, FFAR3 suppresses energy expenditure and produces β-hydroxybutyrate in the liver during starvation. Thus, sympathetic activity is regulated by SCFA-induced FFAR3, thereby maintaining energy balance. 

**Figure 1:**
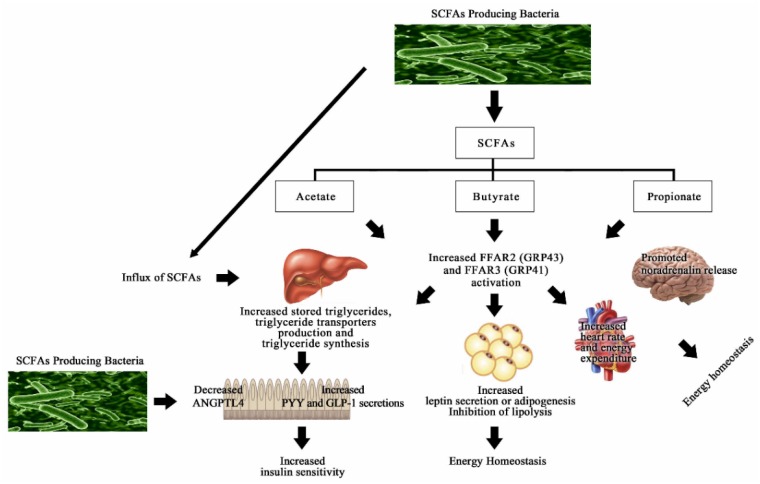
Molecular mechanisms of short-chain fatty acid (SCFA) receptors.

Additional research has indicated that SCFAs are involved in the regulation of hepatic cholesterol synthesis [110,111], as demonstrated via *in vitro* experiments of the liver of germ-free mice. The liver metabolism of germ-free and colonized mice differs considerably, possibly due to the increased influx of SCFAs into the liver of colonized mice [112]. The increased levels of stored triglycerides in the liver and the increased production of the triglyceride transporters were observed in colonized mice. Increased triglyceride synthesis in the liver of colonized mice was associated with reduced expression of fasting-induced adipose factors, or angiopoietin-like 4 (ANGPTL4), in the small intestine. ANGPTL4 inhibits circulating lipoprotein lipase (LPL), which regulates the cellular uptake of triglycerides in adipocytes [113,114]. ANGPTL4 is also a downstream target gene of peroxisome proliferator activated receptors (PPARs), the agonists of which are widely utilized for the treatment of T2DM and CVD [115,116]. PPAR-α mainly plays an important role in hepatic fatty acid oxidation, whereas PPAR-γ constitutes the master regulator of adipogenesis [117]. Moreover, research has indicated that overexpression of ANGPTL4 in the liver leads to decreased activation of LPL and increased plasma triglyceride levels [118]. Interestingly, ANGPTL4 is susceptible to regulation by the gut microbiota [119]. Germ-free ANGPTL4-deficient mice gained considerably more fat mass and body weight compared to colonized mice during high-fat feeding, indicating that ANGPTL4 directly mediates microbial regulation of adiposity in mice [26,120]. Thus, ingestion of SCFAs-producing probiotics could increase influx of SCFAs into the liver, leading to regulation of ANGPTL4 (Figure 1).

SCFA-producing bacteria primarily produce acetate, butyrate, and propionate, which leads to increased FFAR2 and FFAR3 activation. These enhancements of FFAR2 and FFAR3 not only promote noradrenalin release, but also increase heart rate and energy expenditure for energy homeostasis. SCFAs are involved in increased leptin secretion, adipogenesis, and the inhibition of lipolysis in adipose tissues. In the intestine, SCFAs enhance the secretion of PPY and GLP-1. Moreover, an improvement of triglyceride synthesis occurs due to an influx of SCFAs into the liver, which leads to decreased ANGPTL4 activation in the intestines. In addition, SCFA-producing bacteria regulate the suppression of ANGPTL4, an inhibitor of LPL, which promotes increased lipid clearance. 

**Figure 2:**
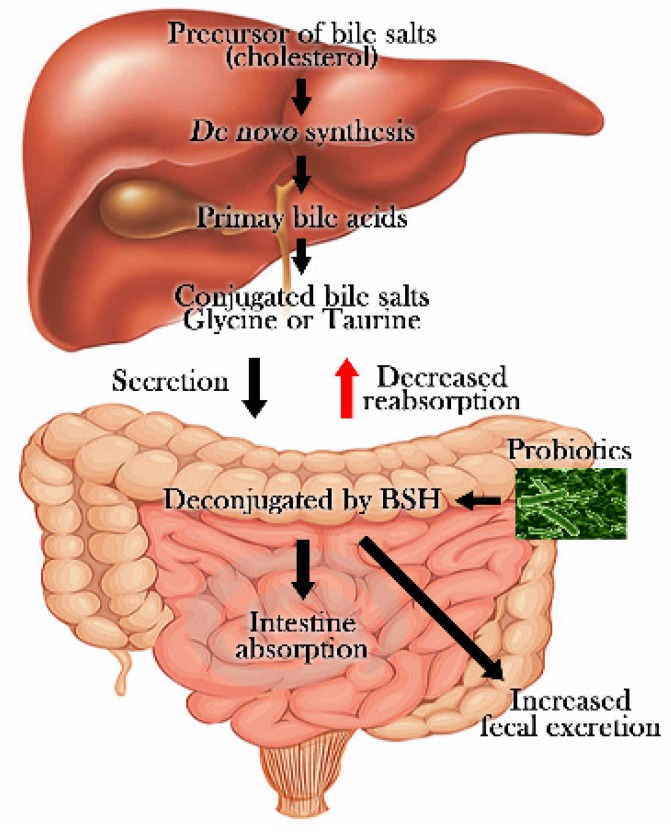
bile-salt hydrolase (BSH) effects on lowering cholesterol by probiotics.

Enzymatic deconjugation of bile acids by bile-salt hydrolase (BSH) has been proposed as an important molecular mechanism in cholesterol-lowering effects. Researchers evaluated BSH’s cholesterol-lowering effect utilizing *Lactobacillus plantarum* 80 and *Lactobacillus reuteri*, whereupon it was shown that the enzyme responsible for bile-salt deconjugation in enterohepatic circulation can be detected in probiotics indigenous to the gastrointestinal tract [53,121]. Bile consists of conjugated bile acids, cholesterol, phospholipids, bile pigment, and electrolytes. Synthesized in the liver, bile is stored at high concentrations in the gallbladder between meals. After food intake, it is released into the duodenum. Bile works as a biological detergent that emulsifies and solubilizes lipids for digestion. BSH catalyzes the hydrolysis of glycine or taurine conjugated primary bile acids to create deconjugated bile acids. The deconjugated bile acids are less soluble and less efficiently reabsorbed than their conjugated counterparts, leading to their elimination in the feces [43,122]. Deconjugation of bile salts can lead to a reduction in serum cholesterol either by increasing the demand for cholesterol for *de novo* synthesis of bile acids to replace those lost in feces or by reducing cholesterol solubility and, thereby, absorption of cholesterol through the intestinal lumen [121,123]. Figure 2 shows the mechanism of enzymatic deconjugation of bile acids by bile-salt hydrolase (BSH).

Cholesterol is utilized as the precursor for synthesis of new conjugated bile acids, and the activation of BSH by probiotics catalyzes primary bile acids to create deconjugated bile acids that are less soluble and less efficiently reabsorbed in the intestine and liver. Decongugated bile acids also contribute to the elimination of cholesterol in the feces.

## 9. Future Prospects

Numerous *in vivo* and/or *in vitro* studies have been conducted utilizing an array of probiotics and/or prebiotics. Key issues in this field are safety and efficacy. Currently, some probiotics (*Lactobacillus, Bifidobacterium*) and prebiotics (inulin, oligofructose) do not require approval from the FDA and are present in our daily dietary intake. Although the safety of probiotics and prebiotics for food application has been confirmed by several legal authorities worldwide, few studies have been conducted regarding incidences of bloating, flatulence, and high osmotic pressure, which can lead to gastrointestinal discomfort [124]. Furthermore, the effects could vary depending on the individual and the type of food containing the prebiotics or probiotics. Probiotics and prebiotics are believed to be safe for oral consumption due to their relatively low capacity to cause adverse effects. However, no standard safety guidelines currently exist for oral administration of probiotics and prebiotics in human cases. Therefore, individual probiotics and prebiotics should be evaluated at specific dosages to ascertain potential adverse reactions.

Although BSH was shown to be beneficial, it may lead to an increase in potentially cytotoxic secondary bile acids in the enterohepatic circulation, which in turn could increase the risk of cholestasis or colorectal cancer [125]. Lithocholic acid (LCA) is a secondary bile acid primarily formed in the intestines by the bacteria. Trauner *et al.* and Beilke *et al.* showed that administration of LCA and its conjugates to animals causes intrahepatic cholestasis. In humans, abnormal bile acid composition, especially an increase in LCA, was found in patients suffering from chronic cholestatic liver disease or cystic fibrosis [126,127]. However, most studies argued mainly for the benefits rather than the adverse effects of BSH from probiotics and/or prebiotics.

The genetic interactions between ingested probiotics and the native intestinal microbes have also constituted a topic of interest. The genetic materials can be exchanged via three mechanisms, including transduction, conjugation, and transformation. The transformation of intestinal microflora by DNA may be enhanced upon the ingestion of bacteria, leading to genetic rearrangements. In addition, the transmission of antibiotic-resistantgenes among beneficial bacteria and harmful pathogens could be associated with a complex microflora colony in the gastrointestinal tract. This transmission can, in turn, lead to the evolution of antibiotic-resistant probiotics and the potential emergence of resistant pathogens [128,129,130,131].

## 10. Conclusions

Metabolic disorders are undoubtedly associated with an increased risk of morbidity and mortality. In our study, we sought to evaluate the effect of probiotics and prebiotics in the context of metabolic disorders. Intestinal microbiota may play an important role in the pathogenesis of T2DM and CVD by influencing body weight, pro-inflammatory activity, and insulin resistance. The scientific community, in general, accepts that the gut microbiota composition and function can be regulated via probiotics and prebiotics. Numerous studies have indicated that probiotics and prebiotics affect T2DM and CVD by changing gut microbiota, regulating insulin signaling, and lowering cholesterol. However, elucidating the interactions between intestinal microbiota and ingested probiotics continues to present a challenge.

Some of the proposed mechanisms and experimental evidence specifically targeting cholesterol-lowering effects remain equivocal. Therefore, more specific and thoroughly designed *in vivo* trials are required to improve our knowledge and eliminate uncertainties. This will, in turn, provide a deeper understanding of the underlying mechanisms and enable us to conduct a more optimal safety assessment prior to the consumption of probiotics and prebiotics by humans. Moreover, no standard safety guidelines currently exist regarding the oral administration of probiotics and prebiotics in human cases. Therefore, individual probiotics and prebiotics should be carefully evaluated in order to determine potential adverse reactions. Future studies are required to increase our understanding of the complex interplay between intestinal and ingested microbiota.

## Figures and Tables

**Table 1:** Characteristics of the included animal studies.

Intervention Type	Name of Pro/Prebiotic Strains	Study Type	Pro/Prebiotic Type and Dose (Per Day)	Duration of Intervention	Outcomes	Parameter without Change	Reference
Probiotics	*Bacillus*, *Lactobacillus*, *Streptococcus*, *Clostridium*, *Saccharomyces*, *Candida*	Rats	Rice bran (10^7^ CFU/g) 30 g/kg	4 weeks	Decreased serum total cholesterol Increase Δ6-desaturase activity and serum arachidonic acid		Fukushima *et al.*, 1999 [74]
Probiotics	*B. lactis* Bb-12, *B. longum* Bb-46	Rats	Buffalo milk yoghurt and soy-yoghurt	4 weeks	Decreased total cholesterol and LDL-C Increasedfecal excretions of bile acids		Abd El-Gawad *et al.*, 2005 [75]
Probiotics	*L. plantarum* PH04	Mice	Human isolate (10^7^ CFU/day)	14 days	Decreased total cholesterol and TG Increased fecal lactic acid bacteria		Nguyen *et al.*, 2007 [76]
Probiotics	*L. acidophilus*, *L. casei*, *L. lactis biovar diacetylactis*	Rats	Dahi 15% (150g/kg)	8 weeks	Decreased glucose intolerance, hyperglycemia, hyperinsulinemia, dyslipidemia and oxidative stress	HDL-C	Yadav *et al.*, 2007 [70]
Probiotics	*L. acidophilus* NCDC14, *L. casei* NCDC19	Rats	Dahi (73 × 10^8^ CFU/g)	28 days	Inhibition of insulin depletion, lipid peroxidation and nitrite formation		Yadav *et al.*, 2008 [69]
Probiotics	*B. animalis lactis* 420	Mice	(10^9^ CFU/day)	6 weeks	Decreased glucose intolerance, tissue inflammation, insulin resistance and secondarily glycaemia		Amar *et al.*, 2011 [48]
Prebiotics	*Inulin*	Rats	5%	4 weeks	Decrease LDL-C, total cholesterol, Liver lipid and TG concentrations Increased HDL-C, and faecal excretions of bile acids		Kim *et al.*, 1998 [77]

Abbreviations: Bifidobacterium (B), lactobacillus (L), streptococcus (S), colony forming units (CFU), tab (tablet), low-density lipoprotein cholesterol (LDL-C), high-density lipoprotein (HDL-C), triglycerides (TG).

**Table 2:** Characteristics of the included human studies.

Intervention Type	Name of Pro/Prebiotic Strains	Study Type	Pro/Prebiotic Type and Dose (Per Day)	Duration of Intervention	Outcomes	Parameter without Change	Reference
Probiotics	*L. acidophilus* L1,	Human	Fermented milk 200 mL/day	4 weeks	Decreased total cholesterol		Anderson *et al.*, 1999 [78]
Probiotics	*B. longum* BL1	Human/Rats	Fermented milk 100 mL/3 ×day	4 weeks	Decreased total cholesterol, LDL-C and TG	HDL-C	Xiao *et al.*, 2003 [79]
Probiotics	*L. acidophilus* LA-1	Human	Freeze-dried Two tablet/day (3 × 10^3^ CFU/tab)	6 weeks		Total cholesterol, HDL-C, LDL-C, TG	Lewis *et al.*, 2005 [73]
Probiotics	*L. fermentum*	Human	Freeze-dried Two tablet/2 × day (2 × 10^9^ CFU/tab)	10 weeks		Total cholesterol, HDL-C, LDL-C, TG liver enzymes	Simons *et al.*, 2006 [80]
Probiotics	*L. casei subsp. casei.*	Human	Yogurt 100 g/day and 200 g/day	6 weeks	Decreased total cholesterol and LDL-C Increased HDL-C		Fabian *et al.*, 2006 [81]
Probiotics	*L. rhamnosus* LC705, *Propionibacterium freudenreichiissp shermaniistrain* JS	Human	Two tablet/day (2 × 10^10^ CFU/tab)	4 weeks		Total cholesterol, HDL-C, LDL-C, TG	Hatakka *et al.*, 2008 [82]
Probiotics	*L. acidophilus* La5, *B. lactis* Bb12	Human	Yogurt 300 g/day (2 × 10^6^ CFU/g)	6 weeks	Decreased total cholesterol and LDL-C	HDL-C, TG	Ejtahed *et al.*, 2011 [22]
Probiotics	*L. acidophilus* La5, *B. lactis* Bb12	Human	Yogurt containing 300 g/day (2 × 10^6^ CFU/g)	6 weeks	Decreased fasting blood glucose levels and HbA_1_c, Increased erythrocyte superoxide dismutase, glutathione peroxidase activities and total antioxidantstatus	Insulin concentration	Ejtahed *et al.*, 2012 [68]
Probiotics	*L. acidophilus*, *L. casei*, *L. rhamnosus*, *L. bulgaricus*, *B. breve*, *B. longum*, *S. thermophiles*	Human	Freeze-dried One tablet/day (14 × 10^9^ CFU/tab)	8 weeks	Decreased serum hs-CRP Increased plasma total GSH Prevention of a rise in fasting plasma glucose		Asemi *et al.*, 2013 [67]
Probiotics Prebiotics	*L. casei*, *L. acidophilus*, *L. rhamnosus*, *L. bulgaricus*, *B. breve*, *B. longum*, *S. thermophiles*, *Fructooligosaccharid-e*	Human	One tablet/day 500 mg/tab	8 weeks	Positive effects on systolic blood pressure	Total cholesterol, LDL-C, HDL-C TG, TG/LDL and LDL/HDL ratios	Mahboobi *et al.*, 2014 [71]
Prebiotics	*Inulin*	Human	Rice-based ready-to-eat cereal (18%)	4 weeks	Decreased total cholesterol and TG Increased breath H2 excretion and fecal lactic acid		Brighenti *et al.*, 1995 [83]
Prebiotics	*Inulin*	Human	One pint of vanilla ice cream (20 g/pint)	3 weeks	Decreased total cholesterol and TG		Causey *et al.*, 2004 [84]

Abbreviations: Bifidobacterium (B), lactobacillus (L), streptococcus (S), colony forming units (CFU), tab (tablet), low-density lipoprotein cholesterol (LDL-C), high-density lipoprotein (HDL-C), triglycerides (TG).
